# Creating value with eHealth: identification of the value proposition with key stakeholders for the resilience navigator app

**DOI:** 10.1186/s12911-020-1088-1

**Published:** 2020-04-27

**Authors:** Aniek Lentferink, Louis Polstra, Austin D’Souza, Hilbrand Oldenhuis, Hugo Velthuijsen, Lisette van Gemert-Pijnen

**Affiliations:** 1 Psychology, Health & Technology, University of Twente, 10 De Zul, Enschede, 7522 NJ The Netherlands; 2 Marian van Os Centre for Entrepreneurship, Hanze University of Applied Sciences, Groningen, The Netherlands

**Keywords:** eHealth development, Stress management, Value specification, Stakeholder involvement, Value proposition design

## Abstract

**Background:**

For a stress-management app to be persuasive and impactful, designers and developers should obtain a clear perspective of the value proposition according to key stakeholders before development. However, this is often not the case. In order to increase the chance of creating an impact by means of the Resilience Navigator app, this study aims to identify key stakeholders and work with them to gain an in-depth understanding of the value proposition of this stress-management app.

**Methods:**

The approach used in this study builds on the approaches taken by Van Limburg et al. and Van Woezik et al. An initial list of stakeholders was identified by means of a literature scan. Stakeholders on this initial list took an online survey to identify key stakeholders with a ranking system. Semi-structured interviews were conducted with a subset of key stakeholders to identify the value proposition using the value proposition canvas as a framework for data collection. Finally, the value proposition was validated by key stakeholders during focus groups.

**Results:**

The key stakeholders identified included employees, employers, participation councils within organisations, HR advisors, product owners, company doctors, and business analysts**.** The interviews produced a list of approximately one hundred values from which fifteen core values were distilled. One example is to take into account time constraints experienced by users during stress periods. In general, the Resilience Navigator app’s main goal is to increase awareness of personal stress levels and causes of stress. In addition, the sub-goal is to increase skills for effective stress management. The focus groups validated the idea that the most important values were reflected in the value proposition and had been appropriately translated into design elements, according to key stakeholders.

**Conclusions:**

A thorough, bottom-up identification and validation of the value proposition for the Resilience Navigator app was obtained, reflecting key stakeholders’ varying ideas on this piece of eHealth technology. The results will facilitate the continued development of the Resilience Navigator app from the value specification phase to the design phase. In the design phase, the remaining assumptions regarding the app’s value proposition should be tested using rapid prototyping.

## Background

Long-term stress has multiple negative consequences for health and well-being [1]. Unfortunately, interventions targeting stress are scarce, as they are often labour intensive to carry out [2, 3]. Self-management via a mobile application may be a solution, and the combination of self-tracking and persuasive eCoaching is seen as a promising platform for preventative measures [4]. Persuasive eCoaching comprises the use of technology to motivate and guide the user through the process of behaviour or attitude change [4]. A scan of the literature and existing stress management apps showed that very few apps harnessed both self-tracking and eCoaching to improve employees’ self-management. Moreover, the few apps that do combine these two components have not integrated them, e.g. by personalising suggestions offered by the automated eCoach based on the self-tracking data [5–7].

Recent decades have seen the launch of many mobile applications that support self-management, including stress management apps [3], many of which have failed [8]. Some of the reasons for these failures are a lack of support for technological problems, a reserved attitude towards eHealth and a lack of awareness of eHealth innovations among end-users and other important stakeholders [9]. In addition, a scan of the literature indicated that successful apps had a well-thought-out business model that included a clear value proposition and revenue model [8]. The use of business modelling for eHealth design has also been introduced as an important issue on the European eHealth policy agenda [10]. In the end, eHealth technology products, just as any other product, have to manage collaboration between different organisations, take additional services, rules and regulations into account, and manage to bring in sufficient revenue streams [11].

The lean start-up movement, a paradigm shift in product and business development, highlights the importance of obtaining evidence that the future product will have added value for its stakeholders as early as possible. In other words, instead of working out the full business plan from the start, developers should liaise with stakeholders to paint a clear picture of the value proposition [12, 13]. Osterwalder et al. define the value proposition as ‘the benefits customers can expect from your products and services’ [14]. Defining the value proposition during the development of eHealth technologies can be valuable in that it sheds light on important factors for improving the market viability of eHealth technology [11], such as the purpose of the technology, i.e. the reason why the technology should be developed, and how it fits into practice at all levels [15]. The value proposition goes beyond the technology’s relevant added value for end-users, on which the well-known approach of human-centred design (HCD) [16] focuses, but also includes how the technology meshes with the technological, organisational and economic needs of all important stakeholders [13, 14].

To clearly define the value proposition, developers must have an understanding of what end-users and other important stakeholders value. Values are the things that stakeholders would like to see reflected and improved or maintained by the eHealth technology [17]. Identifying these values can benefit the development of eHealth technology as well as its implementation in a real-life setting. For the product to actually help stakeholders, developers should have an idea of how stakeholders believe that their values should be translated into actual products and services, i.e. their design requirements.

Although key stakeholder engagement is important during the development of eHealth technology [18, 19], it is often neglected [4]. Key stakeholders are stakeholders with an important say during the design and implementation of eHealth technology, and a lack of stakeholder support can jeopardise its successful uptake. One of the main reasons for a lack of stakeholder engagement is the presumption that maintaining relationships with all stakeholders and comprehensive qualitative data collection and analysis make it a rather time-consuming process [19]. This is exacerbated by the fact that the eHealth domain has a particularly complex stakeholder network [19]. To illustrate this in the context of workplace health promotion, a company doctor (more health-oriented) will have different values than an employer (more economic-oriented), but it is important to acknowledge both sets of values in the development process. Involving stakeholders at an early stage of development can help build a support base for future testing, implementing and disseminating of eHealth technology and could even save time in the end [20].

It is clear that putting effort into identifying the value proposition together with key stakeholders is of major importance for increasing the uptake and impact of an eHealth technology. To our knowledge, no other stress management apps have previously included the identification of key stakeholders and the identification of the value proposition together with key stakeholders in their development process.

This study is part of the development of the Resilience Navigator app, which aims to combine self-tracking and eCoaching to increase stress management skills and resilience among digital screen equipment (DSE) employees. DSE employees are a group considered at risk from stress due to their ability to work from everywhere, obscuring the line between work and private life, and their capacity to process a lot of information in a limited time, making work more intense [21]. Before developing the app, it is important to have a thorough understanding of the value proposition according to key stakeholders, including key stakeholders’ values and the translation and validation of these values into requirements for the eHealth technology. This can improve the app’s uptake and increase its odds of making a difference as the product will be tailored to the stakeholders’ context, needs, and wishes and take into account factors that might influence its market viability [13].

In order to identify the key stakeholders for and the value proposition of the Resilience Navigator app, a synthesize is necessary of methods in the eHealth development domain and business modelling domain. This study’s approach was composed by means of existing and widely accepted methods from these domains [14, 18, 19, 22, 23]. The primary aim of this study is to describe the value-based design process of the Resilience Navigator app by addressing the following research questions:

- Who are the key stakeholders for the Resilience Navigator app?
- What is the value proposition of the Resilience Navigator app according to key stakeholders?

## Methods

**Fig. 1:**
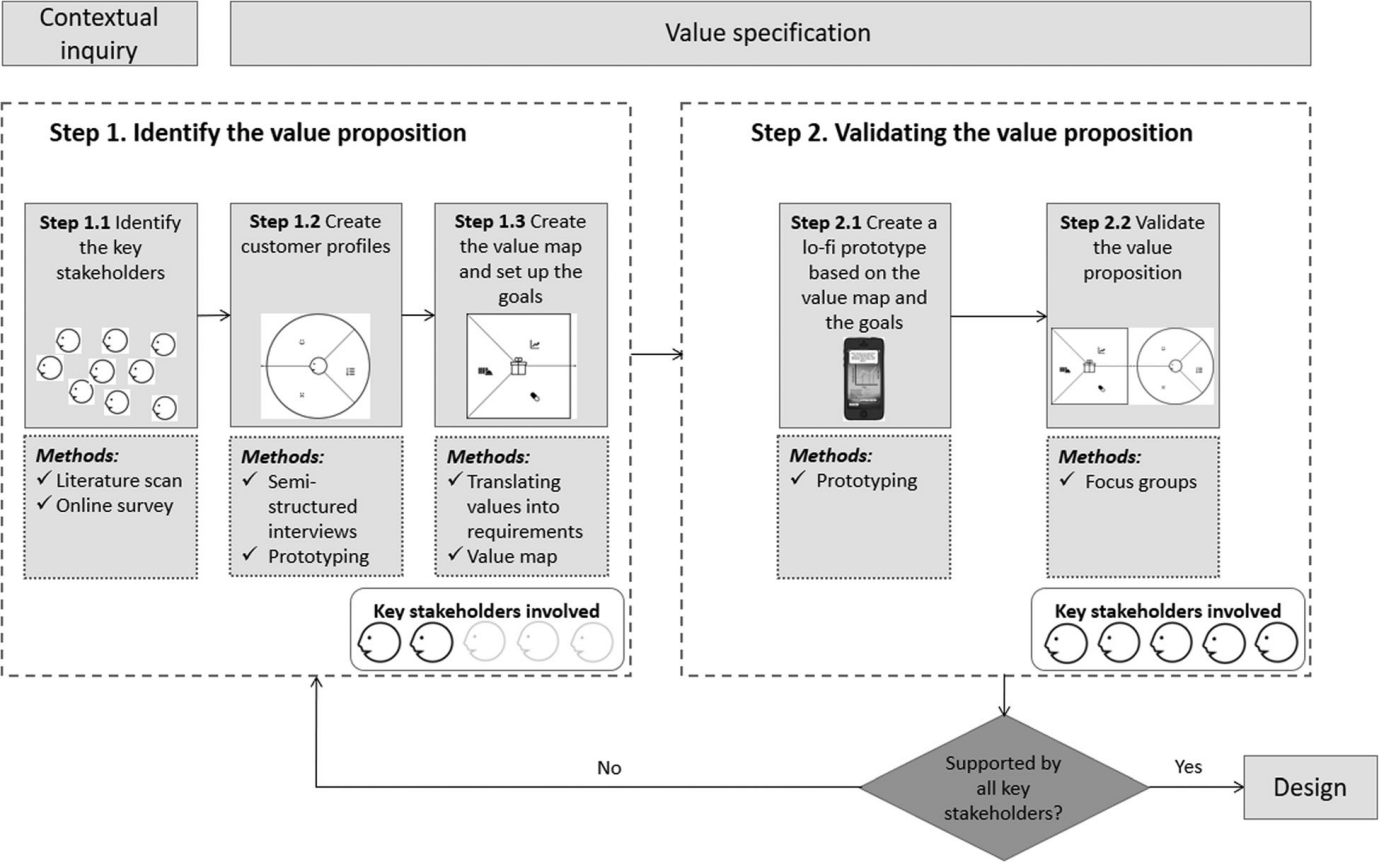
The process map of the research approach. *Note: Permission was granted by* Strategyzer.com *to use the value proposition canvas* [10] *in the figure. The figure was generated using PowerPoint Version 1908 (Microsoft, Redmond, Washington, United States)*

The approach taken in this study, as part of the development of the Resilience Navigator app, builds on the approaches taken by Van Limburg et al. [18] and Van Woezik et al. [19]. Their approaches are a practical translation of the first two phases of the CeHRes (the Centre for eHealth and Wellbeing Research) roadmap. The CeHRes roadmap guides the development and implementation of eHealth technologies through several iterative phases [13]. The first phase of the CeHRes roadmap focuses on the contextual inquiry, which aims to familiarise developers with the context and help identify key stakeholders. In the approach taken by Van Limburg et al. [18] and Van Woezik et al. [19], stakeholders are identified by means of a literature scan and an online survey. The specific steps are described below. The identified key stakeholders are then involved in phase two of the CeHRes roadmap, the value specification phase [19]. The approaches taken by Van Limburg et al. [18] and Van Woezik et al. [19] to identify the values are both sophisticated and structured. For a full understanding of the value proposition, we must also determine how key stakeholders believe that these values should be translated into requirements of the eHealth technology, for which specific steps have been added to the approach described below. In addition, the total value proposition was validated by key stakeholders. An outline of the approach taken can be found in Fig. 1.

To identify and validate the value proposition of the Resilience Navigator app, the well-established value proposition canvas created by Osterwalder et al. was used as a framework for data collection [14]. The value proposition canvas has two sides [14]: (1) the *customer profile,* which fosters an understanding of the customer, or stakeholder, and (2) the *value map,* which shows how the product creates value for stakeholders, i.e. how the values are translated into the design. The customer profiles were identified through conducting semi-structured interviews with a subset of the key stakeholders. The value map was created by translating the values from the customer profile into design requirements. Together, the customer profile and the value map make up the value proposition [14]. Products that manage to fit together the customer profile and the value map have a higher chance of successful development and implementation, resulting in greater impact and improved uptake [14]. Based on the value map, a prototype of the Resilience Navigator app was created. This prototype was pitched during focus groups to key stakeholders to validate the value proposition.

### Key stakeholder identification

#### Literature scan

An initial list of stakeholders was created based on a literature scan that included literature on different types of stakeholder roles [14, 24–26] and earlier research on stakeholder involvement in the development and implementation of workplace health promotion interventions (part of step 1.1 in Fig. 1) [27–34]. The different types of stakeholder roles found in the literature were used as guiding categorisation for identifying stakeholders and ensuring that all stakeholder roles were fulfilled. Stakeholders identified in literature on workplace health promotion were also added to the list. The research team checked the initial list using the following questions: 1) Have the stakeholders been assigned appropriate stakeholder roles?, 2) Are there any stakeholders missing?, 3) Are there any superfluous stakeholders on the list? The research team consisted of researchers in the domain of eHealth development (AL, HO, HV, LVGP), human resources and organisation (LP, HO, HV), and business modelling (HV, ADS). The initial list of stakeholders was discussed in order to increase the chances of creating a complete list of stakeholders.

#### Online survey

An online survey was used to reduce the initial list of stakeholders to a list of key stakeholders (part of step 1.1 in Fig. 1). Potential respondents who could be considered to represent a stakeholder on the initial list were identified via the research team’s personal network. These potential respondents were sent an email with a request to fill in the online survey. This sampling strategy resulted in the involvement of stakeholders from educational institutes, health insurance companies, a labour union, a legal services organisation and companies involved in the development or delivery of eHealth technologies.

The online survey consisted of two main questions and was created with Qualtrics survey software (Qualtrics, Provo, UT) [See Additional file 1 for the online survey]. Question one asked the respondents to name any stakeholders they believed to be missing from the list, while question two asked them to decide on a final list of key stakeholders by means of a ranking system. This ranking system was the stakeholder salience approach, developed by Mitchell, Agle, and Wood [22], which is an often used method for identifying key stakeholders. According to Mitchell and colleagues, stakeholder salience consists of three attributes:

- *Power*: a stakeholder has power when he/she has a direct influence on the development of the eHealth application.
- *Legitimacy*: a stakeholder has legitimacy when he/she HAS to be involved during the development of the eHealth application for legal, moral or contractual reasons.
- *Urgency*: a stakeholder has urgency when he/she imposes requirements that can wait no longer because of time concerns or importance.

When at least one-third of the respondents assigned an attribute to a stakeholder, the attribute was linked to that stakeholder. The rank of each stakeholder was determined by the number of attributes assigned to them.

The research team checked whether all possible stakeholder roles were fulfilled by the key stakeholders identified via the stakeholder salience approach and added any missing stakeholders to the final list. This sub-step was added to the approaches taken by Van Limburg et al. [18] and Van Woezik et al. [19], as including all stakeholder roles in the final key stakeholders list would allow developers to consider the problem at hand from all important perspectives, such as those of the end-user, the buyer, and the recommenders who make a formal recommendation to purchase the product.

### Identification and validation of the value proposition

#### Semi-structured interviews

Semi-structured interviews were conducted to gain an understanding of the stakeholders’ values - i.e. to create customer profiles (step 1.2 in Fig. 1) - and to design a value proposition that reflected what stakeholders want. In other words, the stakeholders determine which values form the foundation for the design of the product.

##### Participants

Semi-structured interviews were conducted with two types of key stakeholders, e.g. DSE employees, to gain a greater understanding of their values. The first type was to represent the most important group of end-users, while the second type was to represent different stakeholder roles. The latter type of stakeholder was chosen by means of maximum variability sampling [35] to increase the chances that it would accurately represent the varied nature of the body of possible respondents, a quality aspect of qualitative research. The two types of key stakeholders, chosen from the full list of identified key stakeholders, are described in the Results section under the heading *Semi-structured interviews – identification of the customer profiles*. Eight respondents per key stakeholder type were invited to participate via the research teams’ personal network, each of whom signed an informed consent form. Key stakeholders from commercial, semi-commercial and non-commercial organisations were involved in anticipation of differences in values regarding the development and implementation of workplace health promotion between those types of organisations. These differences were checked in the analysis stage.

##### Data collection

To gain an understanding of stakeholders, values were identified using the three elements of the customer profile: customer jobs, pains, and gains. The customer jobs reflect the context for which eHealth technology is developed and includes the jobs customers want to achieve, problems that customers are facing and the needs that should be met to successfully perform those jobs. An example is that employees find it hard to say no to requests from colleagues, which increases their workload. In addition, gains reflect the outcomes customers want to achieve, while pains reflect the outcomes customers want to avoid [14]. The three elements were topics on the topic list [see Additional file 2 for the topic list]. The topics of gains and pains dealt specifically with the two overarching components of the Resilience Navigator app, namely self-tracking and persuasive eCoaching. Pains and gains were also questioned in relation to preconditions for the eHealth design to be successful, such as privacy and implementation. In addition, the relative importance of the values was discussed, as it is usually not possible for a single value proposition to take into account all customer jobs, gains, and pains [14]. A persona representing possible end-users [36] was presented at the start of the interview, as stress can be a stigmatising issue. Using a persona enabled respondents to speak from the situation of the persona instead of their own experiences with stress. Interviews took place one-on-one, were conducted by one researcher (AL) and were taped, resulting in a set of audio recordings between 42 and 82 min.

#### Value map

The customer profiles identified during the semi-structured interviews were translated into a value map by drawing a list of goals for the eHealth technology (step 1.3 in Fig. 1). These goals reflected the most important values from the customer profiles. By doing so, a clear focus was defined for the solution. After the goals had been set, the values in the customer profile were translated into requirements. The value map consisted of products and services, gain creators and pain relievers that reflected the most important customer jobs, gains, and pains according to key stakeholders [14].

#### Prototyping

Prototypes were used during the semi-structured interviews (step 1.1 in Fig. 1) and the focus groups (step 2.1 in Fig. 1). During interviews, a lo-fi prototype of the Resilience Navigator app was used to facilitate the value identification process, as stakeholders often struggle to identify values if they have no idea what the technology may come to look like [18]. The lo-fi prototype was shown after customer jobs were identified to gain an initial understanding of the context described by stakeholders for which we are developing an eHealth technology, without immediately focusing on a possible solution [13]. The lo-fi prototype of the Resilience Navigator app was created via Balsamiq.com version 2017 (Balsamiq, Sacramento, California, United States) and was based on a previous scoping review conducted by the authors of this article in order to identify key components of self-tracking and persuasive eCoaching [4].

The lo-fi prototype shown during focus groups, to validate the value proposition, reflected the value proposition of the technology in that the requirements from the value map had been included in the lo-fi prototype.

#### Focus groups

Two focus groups (step 2.2 in Fig. 1) were held with key stakeholders to obtain a consensus on the value proposition of the Resilience Navigator app [37]. The aim of the focus groups was to determine whether key stakeholders believed that the value proposition could lead to a valuable product, i.e. if the value proposition targeted the most important values and if these values had been appropriately translated into requirements.

##### Participants

One respondent of each type of key stakeholder was involved per focus group, resulting in the inclusion of two individuals per type of key stakeholder during the validation of the value proposition. The full list of identified key stakeholders can be found in the Results section under the heading *Online Survey – Key Stakeholder Identification*. The participants in the focus groups were recruited from the research team’s personal network. One focus group was conducted in a non-commercial organisation and one in a semi-commercial organisation. No focus group was organised for a commercial organisation, as the interviews had shown that there were no important value differences between commercial and non-commercial organisations. In addition, the two organisations chosen were already involved in the Resilience Navigator app project, which made the focus groups easier to organise.

##### Data collection

At the start of the focus groups, the goals were presented and the lo-fi prototype was pitched to the respondents. This lent greater clarity to the values addressed in the value proposition and their translation into design elements. Topics for discussion were the goals and topics in the value proposition for which additional information was required or about which no consensus has been reached after the analysis of data from the semi-structured interviews. The choices for the topics were discussed with the research team, based primarily on the interview results. As a final topic, respondents could name other relevant aspects of the design that had not been discussed earlier [see Additional file 3 for the pitch, goals and the topic list]. This enabled respondents to introduce new values or discuss the values or requirements in more detail. In addition to key stakeholders, a moderator (AL) to guide the discussion between stakeholders, and a research assistant to ensure the procedure was followed and to report on non-verbal signals given by stakeholders [37], were present. Immediately after the focus groups, the moderator and the research assistant discussed and reported the main outcomes of the discussion.

### Data analyses

#### Online survey

The results of the survey were uploaded to SPSS version 25. Descriptive statistics, in the form of counts and percentages, were used to identify which attributes the respondents had assigned to the stakeholders on the initial list.

#### Semi-structured interviews

The recordings of the interviews were transcribed and anonymised. All transcriptions were uploaded in Atlas.ti version 8, the statistical software package for qualitative research (Scientific Software Development GmbH, Berlin). The data belonging to each of the two types of key stakeholders were analysed separately with a coding scheme that included the customer jobs, pains, and gains, sensitising concepts from the literature on behaviour change via persuasive technology [38], the previously conducted scoping review [4], and existing methods for stress management [39, 40]. Open coding was used for quotes that did not match any of the sensitising concepts. In addition, the coding scheme was tested and discussed for the consistency of coding by two researchers (AL and LP) by independently coding a subset of the data and discussing any points of disagreement. This resulted in minor adjustments to the interpretation of codes. After several rounds of coding, values were extracted for each code. The relative importance of values according to stakeholders was estimated by coding the pains as ‘acceptable’, ‘unacceptable’ or ‘unknown’ and the gains as ‘essential’, ‘preferred’ or ‘unknown’, based on what was said by the respondents. The level of importance was also discussed for consistency by the two researchers (AL and LP) using a subset of the data. The most important values were eventually added to the customer profiles and translated into requirements in the value map.

#### Focus groups

Focus groups were audiotaped and transcribed within 48 h after they took place. This enabled the transcriber (AL) to attribute quotations to the correct stakeholders and add information about non-verbal signals. The transcripts, notes made by the research assistant during focus groups and the summaries of the discussions between the moderator and research assistant after the focus groups served as input for analyses. Analysis was performed using Atlas.ti version 8 (Scientific Software Development GmbH, Berlin). During the coding process, sensitising concepts were used based on the customer profile, the discussions between the moderator and research assistant, the literature on behavioural change via persuasive technology [38], and the previously conducted scoping review [4]. In addition, open coding was performed for quotations that did not match any of the sensitising concepts.

## Results

### Online survey – stakeholder identification

The literature scan and discussion among the research team resulted in an initial list of 29 stakeholders [See Additional file 4]. 47 potential participants - at an average of more than one participant per stakeholder - were invited to take the survey. Of the 47 participants who were invited, 27 started the survey, while 17 completed it. One respondent reported a missing stakeholder, namely the Ministry of Public Health, Wellbeing and Sports, to which they assigned one attribute. The results of the online survey can be found in Additional file 4.

Due to the absence of any stakeholders that possessed all three attributes, all stakeholders with two attributes were identified as key stakeholders. These were DSE employees, employers, company doctors, participation councils within organisations, and the research team (authors of this article). To guarantee that user needs outweighed the needs of the research team, the latter was given a more distant role during the value specification phase and was therefore not included as a key stakeholder.

In addition, some stakeholders with one attribute were added to the list of key stakeholders, as some of these stakeholders fulfilled roles that stakeholders with two attributes did not (according to the stakeholder’s roles presented by Osterwalder et al. [14]). These stakeholders were HR advisors, business analysts, and product owners. An overview of key stakeholders and their roles can be found in Table 1.

**Table 1:** Stakeholders and their stakeholder roles [14]

Stakeholder	Stakeholder role
DSE employees, employer, participation councils within organizations, company doctors, HR advisors, and product owners	Influencers (Individuals or groups whose opinion might count and whom the decision makers might listen to, even in an informal way)
Business analysts and HR advisors	Recommenders (the people carrying out the search or evaluation process and who make a formal recommendation for or against a purchase)
Employer, company doctor	Economic buyers (The individual or group who controls the budget and makes the actual purchase)
Employer (buyer) and product owner (during development of the product)	Decision makers (The person or group ultimately responsible for the choices in (1) design and (2) purchase decisions. Usually, they have ultimate control over the budget.)
DSE employees	End-users
HR advisors and company doctors (these stakeholders have knowledge about laws and regulations (to prevent from obstruct the process of purchasing a product) and advise which interventions to buy/implement.	Saboteurs (the people and groups who can obstruct or derail the process of searching, evaluating, or purchasing a product.)

### Semi-structured interviews – identification of the customer profiles

DSE employees and HR advisors were selected from the list of key stakeholders in order to help identify the customer profiles. DSE employees were involved because they represented end-users of the eHealth technology, whereas HR advisors represented many different stakeholder roles, such as influencer (someone decision makers may listen to) and recommender (someone who carries out the search or evaluation process for workplace interventions). The participants worked at commercial (n = 5), semi-commercial (*n* = 6), and non-commercial (*n* = 5) organisations. Seven of the respondents were male and nine were female. Ages ranged from 27 to 61 years.

Although the data were analysed separately per stakeholder type, a single customer profile was created, as the results did not differ much between the two types of key stakeholders. Minor differences are described below.

**Fig. 2:**
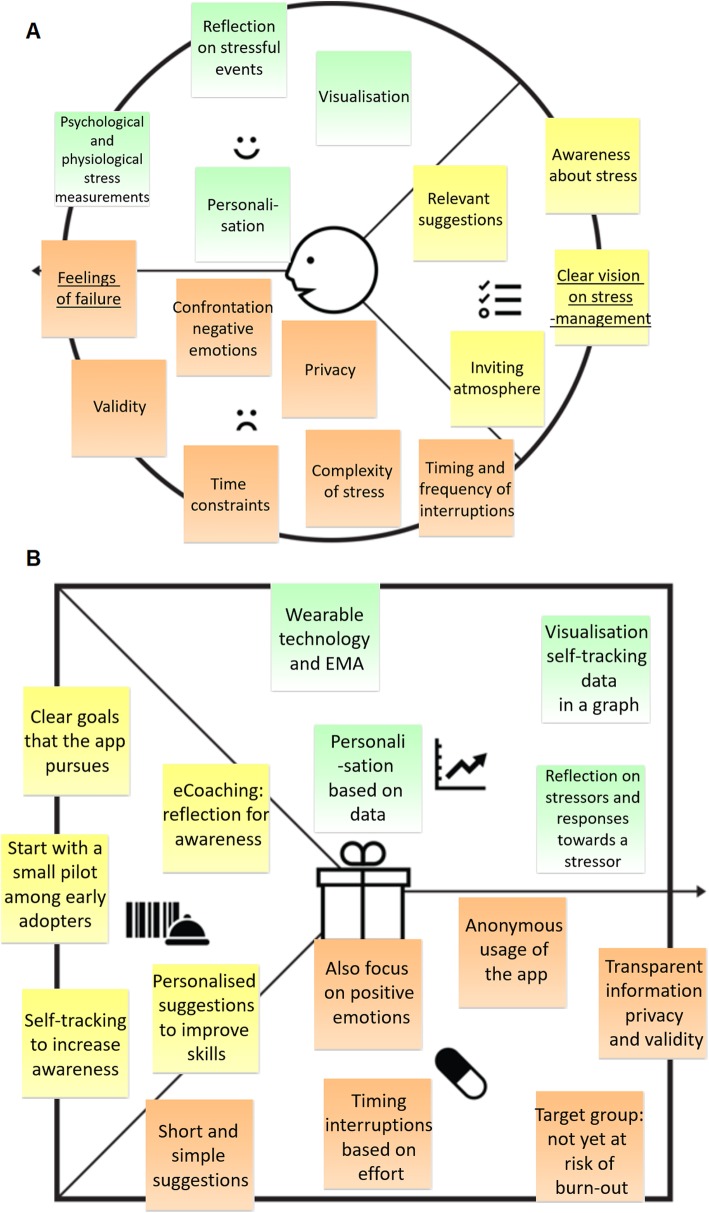
The value proposition of the Resilience Navigator app [14]. *Note: (A) The customer profile including the identified values from the interviews with employees and HR advisors. The outlined values were only mentioned by HR advisors. (B) The value map including the translation of the identified values from the customer profile into requirements for design. Permission was granted by Strategyzer to use the value proposition canvas in the figure. The figure was generated using PowerPoint Version 1908 (Microsoft, Redmond, Washington, United States)*

Each stakeholder group mentioned approximately 100 different values. The customer profile described below contains only the most important values distilled, during the analysis process, from the statements made by the key stakeholders. In the running text, these values are written in *italics.* The customer profile is depicted in Fig. 2a, in which the values only mentioned by HR advisors are outlined. A more in-depth presentation of the results regarding the customer profile has been described elsewhere [41].

#### Customer jobs

One of the first necessities mentioned by key stakeholders was *awareness* of one’s personal stress situation. “I think many people feel a need for it, that bit of awareness. What happened? When and why did it happen? Does it affect ….? [..] Being aware of your stress levels gives you a sense of control, a way to deal with it by yourself”” (HR advisor #1). In addition to awareness, stakeholders highlighted that they would like to receive *relevant suggestions* as to what they can do about stress. On top of that, it was deemed important to *foster an inviting atmosphere in the organisation* that encourages people to take action against stress. Specifically, HR advisors believed it was necessary for organisations to develop a *clear vision on stress management in their health and safety policy*. “I think that you should carefully look into your reasons for implementing a particular measure. What role does it play in the big picture? How do we want to deal with employability and how can we support employees in this? I think you need a good story, a proper vision [..]” (HR advisor #3).

#### Gains

One of the most prominent positive aspects mentioned was *obtaining awareness about the personal level of stress and causes of stress*. In addition, respondents believed that *self-tracking of both physiological* (e.g., elevated heart rate) *and psychological* (e.g., perceived stress) measures of stress would be interesting. Respondents expected that it would be helpful for the awareness process to have *a moment of reflection on the stress* experienced throughout a particular period of time. Similarly, they highlighted the importance of *visualisation* of the self-tracking data: “You are shown an overview of your heart rate throughout the week and you see all these peaks. Hey, was it that day? That’s right, I was very busy at that time.” (employee #1). In addition, an important gain mentioned was *personalisation*. “I think the more you personalise it, the more users feel attracted and addressed by it and the more value such a recommendation would have” (HR advisor #4).

#### Pains

HR advisors specifically expected *feelings of failure* to represent a significant barrier when it comes to motivating employees to do something about stress. “It puts people in a vulnerable position. Why do you experience problems at work while others with exactly the same tasks do not? It’s something people notice, and I think it would be a big pain point for everyone” (HR advisor #8). Also, the end-user should be willing *to face negative emotions* when self-tracking stress. “Depending on what you carry with you, looking within may be very confronting. [ …] You may see something you do not want to see, so you choose not to look within and stay unhappy” (employee #4). Specific pains expected for stress management via wearable technology and smartphone applications are *privacy* and *validity*. Stakeholders deemed it important to thoroughly communicate to users what they can expect from the design for the sake of expectation management. Also, they raised the question as to whether coaching via a smartphone application would be able to deal with the *complexity of stress*. Two other, much-debated pain points were the *timing and frequency* of reminders given by a smartphone application. On the one hand, respondents believed that notifications sent during stress periods would increase awareness, while on the other hand, inconvenient or excessive messages could spark annoyance. This can also be linked to expected *time constraints* among employees with high levels of stress, as it is important that the eHealth technology should fit into their busy days.

### The value map

Based on the customer profile, a value map was created that describes the products and services, gain creators, and pain relievers that represent specific translations of the values from the customer profile to design elements, i.e. the requirements. The related values from the customer profile are shown *in italics* in the text below. Together, the customer profile and the value map make up the value proposition of the Resilience Navigator app. The value proposition is depicted in Fig. 2, with Fig. 2b representing the value map.

#### Products and services

To provide a *clear vision* on how the intervention should be *embedded within the health and safety policy* of an organisation, the intervention’s goals were formulated on the basis of the most important values. Its main goal is to ‘increase *the awareness* of the personal *stress* levels and causes of stress via a smartphone application for DSE employees who do not yet belong to the group at risk of burn-out’. This is also why the eCoach’s main task will be to guide the user through the process of gaining awareness via reflection (customer job *awareness of stress* and gain *reflection on stressful events*). For the reasoning behind the target group selection, please refer to the section on pain relievers.

The sub-goal of the intervention is to ‘improve skills for effective stress management among DSE employees who do not yet belong to the group at risk of burn-out’. Improving skills was chosen as the sub-goal because the respondents indicated that they would like to receive guidance via *personally relevant suggestions* as to what to do about their perceived stress.

The implementation of the app should start with a small pilot developed for a small number of early adopters of eHealth technologies within the organisation to collect initial positive experiences with the design. This could contribute to creating *an inviting atmosphere within the organisation* for stress management using a smartphone application.

#### Gain creators

Seeing as *physiological and psychological measures* are considered interesting, both wearable technology for physiological measures (e.g. elevated heart rate) and ecological momentary assessments, i.e. short questionnaires filled in during the day for psychological measures (e.g. experienced stress) will be used to collect data on stress. The collected self-tracking data will be visualised in a graph (gain *visualisation).* The eCoaching component will focus on reflecting on physical, mental and emotional responses towards a stressor as well as the causes of stress (gain *reflection on the moment of stress* and *awareness of stress)*. Together with the user input on reflective questions, this continuous stream of self-tracking data can be used to provide personalised suggestions (gain *relevant suggestions*).

#### Pain relievers

To acknowledge the *complexity of stress*, DSE employees who are not yet at risk of burn-out are chosen as the target group. In addition, automated systems allow DSE employees to remain anonymous (pain *feelings of failure*).

The application will also focus on positive emotions to avoid emphasising *negative emotions*. Due to *time constraints*, short and simple suggestions will be provided. With regard to the pain points *privacy* and *validity*, the user will receive transparent information regarding these two aspects. As for *appropriate timing and frequency*, and *awareness* being the main focus of the design, respondents will receive a notification during stressful moments. When more effort is required from the respondent, such as reflecting on or responding to a suggestion, messages will be sent during natural breaks (start of the day, lunch break etc.).

### The lo-fi prototype

**Fig. 3:**
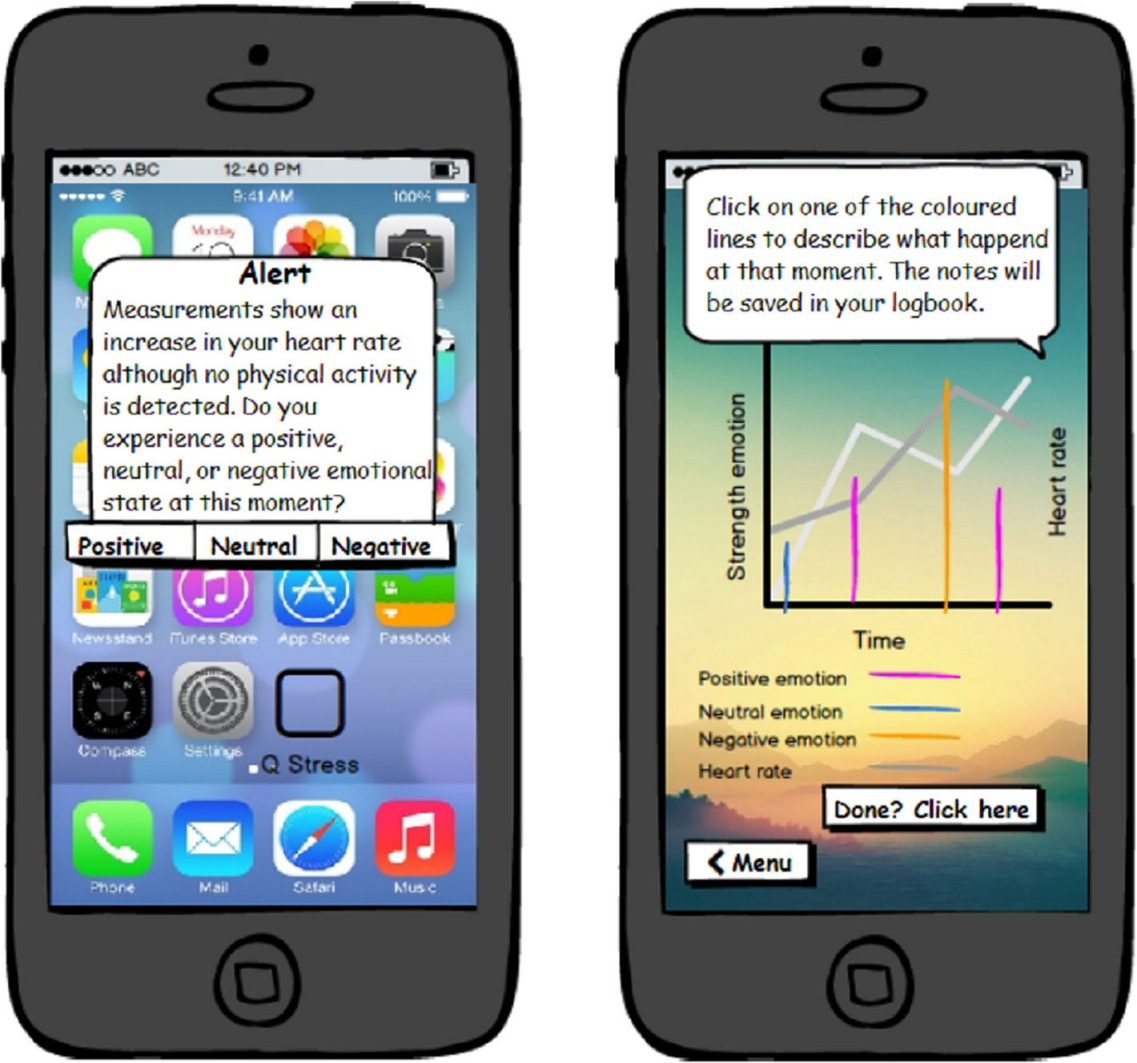
Mock-ups of the Resilience Navigator app using Balsamiq.com version 2017 (Balsamiq, Sacramento, California, United States). Permission was granted by Balsamiq.com to use the mock-ups in this figure

Almost all requirements in the value map were included in the lo-fi prototype for validating the value proposition during focus groups. The requirements *self-tracking to increase awareness* and *eCoaching: reflection for awareness* were given the highest priority during the creation of the lo-fi prototype. These requirements reflected the most important values identified in the customer profile and the app's main goal. The translation of these requirements into the lo-fi prototype can be observed in Fig. 3. The first screenshot reflects the products and services *self-tracking to increase awareness* and the second screenshot reflects the gain creator *reflection on self-tracking data with the eCoach*. The lo-fi prototype was explained orally during the focus groups.

The following requirements were not included in the lo-fi prototype: *(1) transparent information on privacy and validity, (2) the target group: not yet at risk of burn-out, (3) clear goals that the app pursues, and (4) start with a small pilot among early adopters*. These requirements focus on the implementation of the eHealth technology and are somewhat difficult to visualise in the lo-fi prototype. The implementation requirements that were a topic of discussion during focus groups were verbalised in the pitch. These were (1) *the target group* and (2) *clear goals that the app pursues*.

### Focus groups - validation of the value proposition

Two focus groups were held. One respondent of each type of key stakeholder was present per focus group. Participants were aged between 32 and 63 years, with half being male and half female. The topics, based on the interview data, were: (1) the goals and target group of the Resilience Navigator app, (2) reflection as the main focus of automated eCoaching, (3) timing and frequency of messages, and (4) embedding in organisations’ health and safety policy. These topics also emerged as themes during data analysis, as did the app’s marketing strategy and self-tracking component.

Goals and target group of the Resilience Navigator app.

Focus group participants mainly shared the idea that the intervention’s primary goal should be to increase awareness of personal stress levels and causes of stress. However, they also believed that the effectiveness of automated eCoaching decreased as people experienced more stress. Although participants believed that a combination of automated eCoaching and human coaching would positively affect adherence to the system, they also acknowledged the advantage of improving stress management anonymously. “Look, there is of course also a group that really, well, drops out and only starts taking action afterwards. Such an app could maybe help this group take action at an earlier stage” (product owner, focus group #2).

#### Reflection as the main focus for automated eCoaching

Reflection on the self-tracking data was perceived as an important aspect of stress management. “I like asking them: ‘when things are not going well, do you ever take a moment to consider how you actually feel?” (company doctor, focus group #2). Helping users interpret their self-tracking data is also an important component of the eCoaching component in the Resilience Navigator app.

#### Timing and frequency of messages

Expectations regarding the proper timing and frequency of notifications differed among the focus group participants, but the group did agree that users should have some level of control over self-tracking and eCoaching messages. In addition, the participants believed that users may be willing to perform intensive self-tracking for a certain period of time, but would feel the need to scale back after a while. Some participants also saw the advantage of eCoaching in the moment: “What can you do to influence stress? If the stress is linked to a particular activity, location or time period, that’s where or when you should intervene. In my opinion, that’s absolutely crucial” (HR advisor, focus group #1). In addition, participants believed that a push notification about a bodily response to stress might already prompt small, on-the-spot behavioural changes. Participants believed that the tolerance to push notifications will be improved when little effort is required to use the app and when advantages have been experienced from using the app.

#### Health and safety policy embedment

Participants agree that the app should not stand alone and should mesh with organisations’ health and safety policies. It should be an integral, fixed part of an organisation’s policy, rather than a short-term hype. Participants expressed the concern that organisations might lose sight of the bigger picture when employees start working on stress by themselves. “As a supervisor, I would start feeling a bit alienated from my employees when they do not open up about this topic. I would like to say to them: ‘talk with me because I think we have something to discuss here’.” (focus group #1, HR advisor).

#### Self-tracking

Focus group participants shared several comments regarding the self-tracking element. First, it is important to realise that experiencing minor, yet frequent moments of stress does not necessarily translate to a bad day overall. Participants believed that it is important to guide users by monitoring their situation from a helicopter view. Still, they also saw advantages in tracking brief moments of stress. Participants believed that when brief moments of stress occur frequently, they could significantly affect an employee’s overall state of mind.

#### Marketing strategy

Participants believed that it is hard to motivate DSE employees not yet at risk of burn-out to do something about stress in the preventative phase. One way to motivate these employees could be to create a marketing strategy that focuses on the gains (increased productivity or higher energy levels during working hours) instead of the pains of stress (better stress management). “I think a lot of people would also want to use it because it brings something positive to their lives. All these modern-day gizmos and gadgets rarely actually solve a problem, a big problem. Most often, they are only a small piece of the puzzle. If you want to market the product at some point, what does it give people? It gives them a shot of positivity!” (business analyst, focus group #2).

## Discussion

This paper aimed to identify key stakeholders for and the value proposition of the Resilience Navigator app, according to its key stakeholders. With the approach used in this study, we were able to identify the value proposition of the Resilience Navigator app in a thorough, bottom-up fashion. The key stakeholders identified were DSE employees, employers, participation councils within organisations, HR advisors, product owners, company doctors and business analysts**.** Together with the employees and HR advisors, we identified a rich list of approximately one hundred different values, which were condensed into a set of the fifteen most important values that reflected personal values (e.g., increase awareness of stress levels and causes of stress), contextual values (e.g., take time constraints experienced by users in stressful situation into account), management values (e.g., formulate a clear vision of stress management within organisations), technological values (e.g., personalisation of the application), and legal values (e.g., privacy). These values were translated into actual design elements, i.e. the requirements. The identified value proposition, including the values and requirements, can be found in Fig. 2. This value proposition was translated into a prototype, before being validated by key stakeholders in focus groups. As a result, we obtained evidence that key stakeholders generally supported the value proposition, with its fifteen most important values, and that these values had adequately been translated into design elements according to key stakeholders.

This study was the first to identify the value proposition of a stress-management app according to its key stakeholders. Understanding what key stakeholders consider the most important values and translating these values into requirements increases the chance of developing successful eHealth technology [13]. The value proposition of the Resilience Navigator app identified in this study serves as a strong foundation for the further development and implementation of the app. This is further emphasised by the fact that earlier research aligns with the main goals identified for the Resilience Navigator app. During focus groups, key stakeholders agreed that the app’s main goal is to increase awareness of personal stress levels and causes of stress. According to literature, awareness of one’s current situation is an important first step in the process of behavioural change [42]. Knowing what situations cause stress can help locate meaningful starting points for change. The results of a systematic review indicate that using a mental health app to monitor your mood can increase self-awareness and could reduce depressive symptoms [43]. According to the key stakeholders, the app’s sub-goal is to improve skills for effective stress management. Earlier research supports the idea that using an app to give users short, simple suggestions can decrease stress [44]. Key stakeholders believe that a stress-management application is suitable for DSE employees who do not yet belong to the group at risk of burn-out. Respondents believe that employees experiencing higher levels of stress require more than automated coaching alone. This is in line with advice given in the EU compass for action on mental health and well-being to take preventative measures to prevent employees from becoming at risk of burn-out [45].

Although the results may also inform the development of other stress management apps, it should be kept in mind that value propositions are highly context-dependent. It is therefore recommended to identify key stakeholders for and the value proposition of future stress management apps separately. When comparable results are found, this may indicate that the value proposition includes values and requirements that apply to all stress management apps in general.

Some specific advantages that we experienced as a result of the approach described in this article are worth mentioning. Firstly, our in-depth mapping of stakeholder understanding, using topics from the value proposition canvas [14], gave us a lot of valuable insights into the personal and professional contexts of the key stakeholders. Focusing on the problem first, rather than leaping straight to a solution, can help developers avoid sinking time and money into developing a product that end-users will not use [46]. Secondly, a feedback loop was included by having the value proposition validated by key stakeholders. Results from the focus groups show that key stakeholders generally supported the value proposition of the Resilience Navigator app. This provided us with some proof that we had successfully identified the value proposition of the app according to key stakeholders. Thirdly, we took a phased approach to involving the key stakeholders. Including all key stakeholders from the start would have required the inclusion of many respondents and separate data analysis per stakeholder [14], resulting in a time-consuming process. In this study, the researchers selected two types of key stakeholders based on maximum variety sampling [35]. This method aimed to ensure that the two selected types of key stakeholders would largely represent the variety in stakeholder roles. If, during the focus groups, the key stakeholders had not been on the same page, we would have had to take a step back and create separate customer profiles for key stakeholders that disagreed (see the loop in Fig. 1).

Some limitations of the study should also be mentioned. Firstly, we were not always able to discuss values in great detail during the interviews, which meant that we had to make assumptions about the level of importance of various values during the data analysis process. We tried to remedy this issue by having two researchers (AL and LP) analyse and discuss any assumptions that had to be made for a subset of the data in order to reach a consensus. In addition, the feedback loop validated that the most important values were reflected in the value proposition according to all key stakeholders.

Secondly, the use of personas and prototyping could have directed the cognitive process of the participants towards the identification of values based on their expectations instead of their experiences, while the latter are probably more closely related to real-life situations. However, we did not get the impression that these instruments influenced the participants too much, as their statements also included personal experiences with stress and using eHealth solutions. To be sure, we might decide to test the value proposition in real life through hi-fi prototyping, so as to provide the necessary evidence that experiences match the participants’ expectations.

Finally, the identified set of values in the value proposition requires continuous updating. In our study, new values were identified, and new questions arose during the validation of the value proposition. Values are not stable data and are highly dependent on the context in which they are specified, indicating an iterative and dynamic process that is never really complete. For this reason, it is advised to continue the development of eHealth to the design phase when some level of evidence that key stakeholders generally support the value proposition is obtained, e.g. by using the build-measure-learn theory from the lean start-up movement [12]. This theory suggests testing the assumptions of the value proposition with key stakeholders using rapid prototyping. For the Resilience Navigator app, such an assumption would be the testing of the timing and frequency of messages.

Based on our experiences, we believe that the approach used in this study can help identify key stakeholders for and the value proposition of other eHealth technologies, and can even have added value beyond the context of eHealth. To validate whether this is true, future research should test whether the approach yields a value proposition that is generally supported by key stakeholders in other cases as well.

Although the approach described here has only been tested with one case study, we believe it can have specific added value for design cases characterised by a complex set of stakeholders with different agendas [19]. In our case, for instance, employers and employees could have conflicting interests. In addition, this approach can be particularly helpful for cases in which the added value of using technology is not immediately apparent, e.g. self-management for the prevention of stress among employees who may not yet experience stress as a problem. Using the value proposition can clearly pinpoint the added value from multiple perspectives, as reflected by the different perspectives represented among the values identified in this study. Moreover, identifying a value proposition guides the process of translating these values into the design by prompting developers to explore the context in which the technology will be used [14].

## Conclusions

This study aimed at identifying the key stakeholders for and the value proposition of the Resilience Navigator app according to key stakeholders. A value proposition was defined for the Resilience Navigator app, based heavily on the needs and wishes of key stakeholders. Important values and the translation of these values into actual design elements were identified and validated among key stakeholders. Key stakeholders agreed that the Resilience Navigator app’s main goal was to increase awareness of personal stress levels and causes of stress, while its sub-goal was to improve skills for effective stress management. The app is expected to contribute to stress management among DSE employees who do not yet belong to the group at risk of burn-out. The value proposition identified by key stakeholders allows us to take the development of the Resilience Navigator app from the value specification phase to the design phase. In this design phase, assumptions of the value proposition should be tested by means of rapid prototyping. In addition, the approach used in this study could also be of added value during the development of other eHealth technologies. If comparable values and requirements are identified using this approach during the development of other stress management apps, this may result in the determination of values and requirements that apply to all stress management apps in general.

## Supplementary information

**Additional file 1.** Online survey used for the stakeholder identification. This additional file includes the content of the online survey that was send to participants that contributed to the identification of the key stakeholders.

**Additional file 2.** Topic list used during the semi-structured interviews. This additional file includes the topic list that was used during the semi-structured interviews.

**Additional file 3.** Pitch, main goals and topic list used during the focus groups. This additional file includes the pitch of the prototype, the main goals the product pursues, both based on the value proposition, and the topic list that was used during the focus groups.

**Additional file 4.** Results of the online survey for the stakeholder identification. This additional file includes the results of the online survey for the identification of the key stakeholders. In this online survey, each stakeholder on the initial list was ranked based on three attributes, namely ‘power’, ‘legitimacy’ and ‘urgency’. The frequencies and percentages are shown for each stakeholder on these three attributes.

## Data Availability

The datasets used and/or analysed during the current study are not publicly available due to the fact that no consent has been provided by the respondents of this study before data collection took place. Datasets are available via the corresponding author on reasonable request.

## Abbreviations

DSE employees: employees working with digital screen equipment
The CeHRes roadmap: The Centre for eHealth and Wellbeing Research roadmap
