# Supplementary material for: Creating value with eHealth: identification of the value proposition with key stakeholders for the resilience navigator app
Source: BMC Med Inform Decis Mak. 2020 Apr 27;20:76. doi: 10.1186/s12911-020-1088-1 (PMC7184708; doi:10.1186/s12911-020-1088-1)
Supplement: Supplementary file 1 — Additional file 1. Online survey used for the stakeholder identification. This additional file includes the content of the online survey that was send to participants that contributed to the identification of the key stakeholders. [file 12911_2020_1088_MOESM1_ESM.pdf]

Additional file 1 - Online survey used for the stakeholder identification.

Introduction

Thank you for taking the time to complete our questionnaire. This will take about 15 minutes.

If you have read the mail carefully, you can skip the following two paragraphs:

We are currently developing an eHealth application for the employee (Hanze University of Applied Sciences - University of Twente). eHealth includes all kinds of programs for promoting or supporting health via technology (in our case via a smartphone). The smartphone application will generally consist of a combination of self-tracking devices and an eCoach. Examples of self-tracking devices are the Fitbit Zip, Misfit Shine or Apple Watch. These types of devices can objectively map lifestyle factors such as physical activity and sleep of the user. On the basis of information obtained via self-tracking devices, a fully automatic eCoach (without the involvement of a human coach) can give the user personally relevant feedback at the right moment in time. The application aims to promote a healthy lifestyle among employees. We find it is important that the application fits well with the values and wishes of stakeholders in the future.

We would like to involve you as a stakeholder in the research. In order to identify the values and wishes of stakeholders, we are looking for the most important stakeholders to involve during the development of the eHealth application by means of a questionnaire. The next step in the study will be to conduct interviews with key stakeholders identified as a result of the questionnaire.

The list of stakeholders is possibly not comprehensive. There is room in the questionnaire to note stakeholders that you believe are missing *.

** The questionnaire data will be processed anonymously. When we report on the study, we will not present individual results, but only results at group level. The study was approved by the Ethics Committee of the University of Twente.*

Thank you for participating in this survey!

Het Quantified Self @Work team:

prof. dr. Lisette van Gemert-Pijnen
dr. Hugo Velthuijsen
dr. Louis Polstra
dr. Martijn de Groot
dr. Hilbrand Oldenhuis
Aniek Lentferink, MSc

Question 1. To which stakeholder(s) from the list below do you think you have the best fit? Enter in the text space below the number of years that you have been active in the professional field of the relevant stakeholder.

Below, you will find a short description of each stakeholder.

1. Accountmanager
2. Labour and organisation specialist
3. Occupational health and safety services
4. Dutch Data Protection Authority
5. Company doctor
6. Business analyst / innovation expert
7. Central unions
8. Coronel Institute for Labour and Health
9. Data storage specialist
10. eHealth specialist
11. Aesthetics specialist
12. Health behavior specialist
13. Graphic designer
14. HR manager
15. Inspectorate of Social Affairs and Employment
16. Labour lawyer
17. Lifestyle coach
18. Maintenance specialist (maintenance: the long-term use of the application)
19. Marketing department
20. Participation council within an organization
21. Research team
22. Organisation in eHealth design
23. Programmer / software developer
24. Product owner
25. Self-tracking device developer
26. Usability specialist
27. Employer / supervisor
28. Employee
29. Health insurer

Stakeholder	Description
**Accountmanager**	Account managers are contact persons within organizations for customers. They maintain relationships with customers and ensure the purchase of the product.
**Labour and organisation specialist**	The labour and organisation specialist is concerned with issues about work and health, stress, sustainable employability and vitality, work pressure, fitness for work and occupational health and absenteeism.
**Occupational health and safety services**	An occupational health and safety services is an adviser and partner in the field of labour and health and offers service during the event of absenteeism, limiting the duration of absenteeism and preventing absenteeism.
**Dutch Data Protection Authority**	The Dutch Data Protection Authority is an independent body that supervises, advises, informs, and provides information about the use of personal data in accordance with the General Data Protection Regulation (GDPR).
**Company doctor**	A company doctor is primarily a doctor who decides whether there is a medical explanation for an employee not to fulfil his employment contract. The company doctor also formulates labour advice (limitation in hours or tasks) and in some cases the company doctor plays a role in the field of prevention and advice to the organisation.
**Business analyst / innovation expert**	A business analyst and innovation expert have in common that they give advice on formulating requirements for innovations and have an eye for the chances of success of the innovation on the market.
**Central unions**	Central unions negotiate about working conditions with the employer on behalf of the employees. Think of wages, the collective labour agreement and, for example, working hours and working conditions.
**Coronel Institute for Labor and Health**	An institute that focuses on the broad field of labour and health. The activities of the department are focused on the health and health care of the employee with the aim of optimal prevention of occupational diseases and promoting labour participation.
**Data storage specialist**	A data storage specialist has knowledge about the safe storage and easy retrieval of data.
**eHealth specialist**	An eHealth specialist has knowledge about the use of technology to support or improve health and healthcare.
**Aesthetics specialist**	A specialist with knowledge of aesthetics and pays attention during development, for example, on decoration, edge demarcation, symmetry, light, colour and harmony.
**Health behavior specialist**	A health behaviour specialist has knowledge about positively influencing / promoting behaviour for the benefit of health.

**Graphic designer**	The graphic designer develops the graphic design of the product, including layout, typography (fonts, sizes, column width, line spacing, etc.), the use of colour and the illustrations and photos.
**HR manager**	The human resources manager determines the personnel policy of a company and ensures that it is implemented. The HR manager is the link between management and employees.
**Inspectorate of Social Affairs and Employment**	The inspectorate is working on a supervision policy that prevents accidents at work, occupational diseases, absenteeism and incapacity for work as much as possible. The Inspectorate does this on the basis of risk and environmental analyses.
**Labour lawyer**	The labour lawyer advises organisations on matters such as employment contracts and conditions, layoffs, reorganisations, sickness and disability. In addition, the labour lawyer litigates in the event of conflicts.
**Lifestyle coach**	A lifestyle coach guides people in the taking on and maintaining a healthy lifestyle.
**Maintenance specialist (maintenance: the long-term use of the application)**	A maintenance specialist has knowledge about how an application remains interesting for the user for a longer period of time.
**Marketing department**	The marketing department is responsible for the promotion of the eHealth application.
**Participation council within an organization**	Participation councils are bodies made up of employees and influence working conditions, working relationships and the organization.
**Research team**	The research team are the project leaders for the development of the eHealth application. The researchers have backgrounds in eHealth, innovation, ICT, labour participation, Quantified Self, psychology and prevention and public health.
**Organisation in eHealth design**	Organisations in eHealth design develop technology to support or improve health and healthcare.
**Programmer / software developer**	A programmer is a person who writes computer software.
**Product owner**	Organisation that invests financially in the development of the eHealth application and offers / sells the eHealth application to interested parties.
**Self-tracking device developer**	Organisations that develop self-tracking devices such as Fitbit, Jawbone, Misfit and Garmin.
**Usability specialist**	A usability specialist ensures that the system is user-friendly in use.
**Employer / supervisor**	Employer / manager of the organisation within which the eHealth application is distributed among the employees.
**Employee**	Employee of the organisation in which the eHealth application is implemented.
**Health insurer**	Organisations in the provision of health insurances.

Question 2a. Stakeholder ranking

For the stakeholder ranking, the attributes "power", legitimacy "and" urgency "have been selected. The scores on these attributes identify the main stakeholders. The attributes are explained below.

- **Power:** a stakeholder has power when he/she has a direct influence on the development of the eHealth application. An example is an employer who gives a caregiver the assignment to replace regular client meetings with video conversations. The employer is the stakeholder with power.
- **Legitimacy:** a stakeholder has legitimacy when he/she HAS to be involved during the development of the eHealth application due to legally, morally, or contractually reasons. An example of a stakeholder with legitimacy is an organisation who provides a quality mark. Without this quality mark, customers may refuse a product.
- **Urgency:** a stakeholder has urgency when he/she imposes requirements that no longer can wait because of time or importance. An example of stakeholders with urgency are citizens in remote areas who impose that video conversations with doctors become available in acute situations to decrease the risk for mortality.

Please indicate per stakeholder in the table below whether you believe one or more of the above attributes should be assigned to that stakeholder.

In addition, please note any stakeholder that you believe is missing from the stakeholder list presented in the table.

	Power	Legitimacy	Urgency	None (4)	I don't know (5)
1. Accountmanager	-	-	-	-	-
2. Labour and organisation specialist	-	-	-	-	-
3. Occupational health and safety services	-	-	-	-	-
4. Dutch Data Protection Authority	-	-	-	-	-
5. Company doctor	-	-	-	-	-
6. Business analyst / innovation expert	-	-	-	-	-
7. Central unions	-	-	-	-	-
8. Coronel Institute for Labour and Health	-	-	-	-	-
9. Data storage specialist	-	-	-	-	-
10. eHealth specialist	-	-	-	-	-
11. Aesthetics specialist	-	-	-	-	-
12. Health behavior specialist	-	-	-	-	-
13. Graphic designer	-	-	-	-	-
14. HR manager	-	-	-	-	-
15. Inspectorate of Social Affairs and Employment	-	-	-	-	-
16. Labour lawyer	-	-	-	-	-
17. Lifestyle coach	-	-	-	-	-
18. Maintenance specialist (maintenance: the long-term use of the application)	-	-	-	-	-
19. Marketing department	-	-	-	-	-
20. Participation council within an organization	-	-	-	-	-
21. Research team	-	-	-	-	-
22. Organisation in eHealth design	-	-	-	-	-
23. Programmer / software developer	-	-	-	-	-
24. Product owner	-	-	-	-	-
25. Self-tracking device developer	-	-	-	-	-
26. Usability specialist	-	-	-	-	-
27. Employer / supervisor	-	-	-	-	-
28. Employee	-	-	-	-	-
29. Health insurer	-	-	-	-	-

Question 2b. Please note here if you believe any stakeholder is missing from the stakeholder list presented in the table. In addition, please report if this stakeholder has power, legitimacy and/or urgency or none of the attributes during the development of an eHealth application for employees?

____

____

____

____

____

Question 3a. Differences in phases of creating eHealth

We have divided the process of creating the eHealth application for the employee into two phases, namely: design and implementation. **The design phase** is the actual development of the application. **The implementation phase** is the application will be implemented in the daily work setting of employees.

Do you believe that certain stakeholders score differently for the three attributes per phase?

- Yes
- No

Question 3b. Please indicate here to which stakeholders this applies and how these stakeholders differ in attributes per phase.

____

____

____

____

____

Question 4. In the text box below there is room for sharing experiences regarding the completion of the questionnaire.

____

____
