# Supplementary material for: Creating value with eHealth: identification of the value proposition with key stakeholders for the resilience navigator app
Source: BMC Med Inform Decis Mak. 2020 Apr 27;20:76. doi: 10.1186/s12911-020-1088-1 (PMC7184708; doi:10.1186/s12911-020-1088-1)
Supplement: Supplementary file 2 — Additional file 2. Topic list used during the semi-structured interviews. This additional file includes the topic list that was used during the semi-structured interviews. [file 12911_2020_1088_MOESM2_ESM.pdf]

Additional file 2 – Topic list used during the semi-structured interviews.

Table. Topic list of the interviews.

Topic	Question
**Start**	Discuss (without recording) the informed consent form:<br>- Purpose of research & interview<br>- Anonymous processing of data<br>- Monitoring recording & processing<br>- If you do not want to give an answer then you are free to do so (also stop research)<br>- If you have questions, you can ask them at any time<br>- Do you have any questions so far?<br><br>Start recording
**Stress definition**	1. Could you describe what stress entails according to you?<br>a. Symptoms / characteristics
**Customer jobs**	1. From the situation of Miriam/Peter: in which way has stress an influence on:<br>a. Working life<br>b. Private life
	2. 2. What would Miriam / Peter need when it comes to reducing stress?
	3. Which problems would the reduction of stress solve for Miriam/Peter?<br>a. Why is it a problem?<br>b. In what situations?
**Gains**	1. To what extent do you expect that such an application could help reduce stress?
	2. What could be the benefit of using such an application for Miriam / Peter?<br>a. Functional: Could it make a positive contribution to the work? In what way / in which situations?<br>b. Social: Could it make a positive social contribution? In what way / in which situations?<br>c. c. Emotional: Could it make a positive emotional contribution? In what way / in which situations?
	3. What are essential elements that the application should contain for Miriam / Peter to start using the app?
	4. How do you think the application should be designed so that it is appealing for Miriam / Peter to use the app?<br>a. General

	b. Portable sensor technology<br>- Validity of measurements<br>- Manually entering data<br>- Combine data of, for example, agenda, location, email behaviour, causes of stress<br>c. Coaching messages:<br>- Frequency<br>- Time of the day<br>- Appearance of the message<br>- Rewards<br>- Social support. With whom? Colleagues share experiences, etc.?<br>- Goal setting<br>d. Coaching techniques:<br>- Relax exercises<br>- Time management<br>- Cognitive therapy
	5. What preconditions must the app meet so that Miriam / Peter would be satisfied with the app?<br>- How should employees be guided before and during the use of the application?<br>- How would you like to use the app during the day?<br>- Privacy: Problems with data collection? Who may have access to the data?
**Pains**	1. What would be barriers for Miriam / Peter to start working on stress management via the app?<br>a. Functional: Do you expect Miriam / Peter to start using the app? What infringement / influence does usages of the app have on their daily activities?<br>b. Social: What if colleagues know that Miriam / Peter are using the app to reduce work stress?<br>c. Emotional: Do you believe Miriam / Peter would enjoy working on stress management via the app?
	2. What could frustrate Miriam / Peter or experience as a nuisance when using such an application?<br>a. General<br>b. Portable sensor technology<br>- Validity of measurements<br>- Manually entering data<br>- Combine data of, for example, agenda, location, email behaviour, causes of stress

	c. Coaching messages:<br>- Frequency<br>- Time of the day<br>- Appearance of the message<br>- Rewards<br>- Social support. With whom? Colleagues share experiences, etc.?<br>- Goal setting<br>d. Coaching techniques:<br>- Relax exercises<br>- Time management<br>- Cognitive therapy
	3. Could Miriam / Peter also experience disadvantages from using the application for the purpose of reducing stress in the workplace setting? What are the disadvantages?<br>a. Functional: Are there negative consequences for Miriam / Peter by using the application during the work / job?<br>b. Social: Could it contribute negatively in the social field? In what way / in which situations?<br>c. Emotional: Could it contribute negatively on an emotional level? In what way / in which situations?<br>d. Privacy: What is your view on the collection of personal data via portable sensor technology?
**End**	1. Would you use such an application for stress management yourself?<br>Yes/No/Doubt
