# Supplementary material for: Creating value with eHealth: identification of the value proposition with key stakeholders for the resilience navigator app
Source: BMC Med Inform Decis Mak. 2020 Apr 27;20:76. doi: 10.1186/s12911-020-1088-1 (PMC7184708; doi:10.1186/s12911-020-1088-1)
Supplement: Supplementary file 3 — Additional file 3. Pitch, main goals and topic list used during the focus groups. This additional file includes the pitch of the prototype, the main goals the product pursues, both based on the value proposition, and the topic list that was used during the focus groups. [file 12911_2020_1088_MOESM3_ESM.pdf]

Additional file 3 – Pitch, goals and topic list used during focus groups.

Pitch Resilience Navigator app:

How useful would it be if an app helps you to understand your personal stress moments and causes of stress so that you can draw up a personal plan for reducing work stress?

Imagine, you have been sitting for 3 hours at your computer. You have a tight deadline and then all sorts of emails come in with urgent questions for which you actually don't have time... you are frustrated enormously but you ignore the rushed feeling and continue steadily. Then suddenly: "Pling": you receive a notification on your smartphone that the smartwatch has measured an increased heart rate while you are not moving. The app asks whether you are experiencing a positive, negative or neutral emotion at that time. You realize that the increased heart rate is the result of a negative emotion as a result of stress. You remember that the app taught you that short breaks are helpful for concentration levels and stress reduction. Just 5 minutes away from the workplace and a walk will help you to catch your breath and then work on the task again in a concentrated way. At the end of the working day, you will receive a notification again: your heart rate has increased again. Now you realize that a positive emotion as a result of the completed task has caused an increased heart rate. At the end of the day you will see an overview of the experienced emotions and you will be asked to indicate what was the basis of the experienced emotions: what was going on at the time? The app stores the answers in your personal logbook. The next time, the app reminds you that a break helps you to continue working efficiently so you can end the working day with a positive feeling. In your environment, you know people who have dropped out due to high levels of stress. Results on a stress test indicate that you do not yet belong to the risk group for a burn-out, but that your work often entails feelings of stress. In addition, you know that a drop-out due to a burn-out involves a long recovery process. You are happy that there is an app available that can help you anonymously to understand your levels of stress and the causes of the stress. The app helps you gain awareness and coping strategies to do something about it. At this stage, you feel that the step to really go to the manager or company doctor due to your stress levels is too big. That would feel like failure.

The prototype contains two main components: eCoaching and self-tracking. The app is a closed system: no human coaching is involved. [show prototype to give participants an idea of what the intervention might look like]

Work stress definition:

"A condition in which an employee is unable or does not consider himself able to meet the requirements set by the working environment." (Gaillard, 2003)

Main goal of intervention:

Increase the awareness of the personal stress levels and causes of stress via a smartphone application for employees who do not yet belong to the group at risk of burn-out'

Sub-goal intervention:

Improve skills for effective stress management among employees who do not yet belong to the group at risk of burn-out'.

Table. Topic list during focus groups.

Topic	Question	Available time
**Pitch + show prototype**	Explanation 5 minutes<br>Introductory round + inquiries about eHealth experience<br>Prototype 10 minutes<br>5 minutes for all participants to look at the goals and images of the prototype	20 min
**Coaching during reflection and not more than that**	Key words:<br>Reflection important for awareness, focus eCoaching on reflection, prototype example, stress complex, no human coach because of anonymity and scalability.<br>a. What do you expect this approach could or could certainly not yield for stress reduction?<br>b. To what extent do you believe that an employee could do something about stress without the guidance of a humane coach?<br>c. Do you expect that this approach offers sufficient guidance for people who do not yet belong to the group "risk of burnout"?	20 min
**Timing and dose of reminders**	Disturbing employees in the moment can have negative consequences: disturbing during work can cause stress. However, notifying employees during moments of stress can create awareness and could diminish recall problems when answering to the level of stress and causes of stress.	20 min

	- What do you think of the approach of sending reminders during moments of stress?<br>- To what extent do you believe that creating awareness in the moment is important for reducing stress?	
**Suggestion when intervention is not effective for target group**	Because stress is complex and we do not expect the system to be effective for everyone, we think it is important that the end user will receive an additional suggestion how to deal with stress when levels of stress do not decrease after a certain period of time.<br>- What do you think of the suggestion that the system offers when the intervention proves ineffective?<br>- Who could the end user approach?<br>- Disadvantage: suggestion can cost more work / time / money from professionals.	20 min
**alignment with policy**	HR advisers indicated that the intervention should be in line with the health and safety policy within organizations. (Refer to the goals of the intervention)<br>- In which way could these intervention goals be connected to the existing policy?<br>- Respondents to the interviews indicate that the intervention should not be an isolated step in stress reduction within organisation: What should be preliminary or follow-up steps?	20 min
**Closing question**	Are there any topics that you find important and have not yet been discussed?<br><br>With a lot of time left: possibly discussing other points via the value proposition.<br><br>Would you use it in the future / advise the organization to use it?	10 min
**End**	Explanation of next steps in the design process.<br><br>Are there any final questions about this focus group?	10 min
		Total: 120 min

[The topics discussed are all topics of which no consensus was reached during interviews (reflection by eCoach, timing, and suggestion at the end) or insufficient information was obtained (alignment with policy)].
