# Supplementary material for: Creating value with eHealth: identification of the value proposition with key stakeholders for the resilience navigator app
Source: BMC Med Inform Decis Mak. 2020 Apr 27;20:76. doi: 10.1186/s12911-020-1088-1 (PMC7184708; doi:10.1186/s12911-020-1088-1)
Supplement: Supplementary file 4 — Additional file 4. Results of the online survey for the stakeholder identification. This additional file includes the results of the online survey for the identification of the key stakeholders. In this online survey, each stakeholder on the initial list was ranked based on three attributes, namely ‘power’, ‘legitimacy’ and ‘urgency’. The frequencies and percentages are shown for each stakeholder on these three attributes. [file 12911_2020_1088_MOESM4_ESM.pdf]

Additional file 4 – Results of the online survey for the stakeholder identification.

Table. Results of the online survey

Stakeholder	Power	Legitimacy	Urgency	None	I don't know	Nr. of attributes assigned
**1. Accountmanager**	1 (5.9%)	0	0	**10 (58.8%)**	**6 (35.3%)**	**0**
**2. Labour and organisation specialist**	3 (17.7%)	4 (23.5%)	1 (5.9%)	**7 (41.2%)**	3 (17.7%)	0
**3. Health and safety services**	2 (11.8%)	5 (29.4%)	3 (17.7%)	3 (17.7)	4 (23.5%)	0
**4. Dutch Data Protection Authority**	4 (23.5%)	**9 (52.9%)**	2 (11.8%)	3 (17.7%)	4 (23.5%)	1
**5. Company doctor**	3 (17.7%)	**6 (35.3%)**	**7 (41.2%)**	3(17.7%)	3 (17.7%)	2
**6. Business analyst/innovation expert**	2 (11.8%)	**7 (41.2%)**	0	4 (23.5%)	4(23.5%)	1
**7. Central Unions**	5(29.4%)	4 (23.5%)	3 (17.7%)	5 (29.4%)	3 (17.7%)	0
**8. Coronel institute for Labour and Health**	0	3 (17.7%)	1 (5.9%)	4 (23.5%)	**10 (58.8%)**	**0**
**9. Data storage specialist**	3 (17.7%)	3(17.7%)	3 (17.7%)	**7 (41.2%)**	1 (5.9%)	0
**10. Aesthetics specialist**	3 (17.7%)	1 (5.9%)	0	**7 (41.2%)**	6 (35.3%)	0
**11. eHealth specialist**	4 (23.5%)	**7 (41.2%)**	4 (23.5%)	2 (11.8%)	3 (17.7%)	1
**12. Health behaviour specialist**	3 (17.7%)	5 (29.4%)	2 (11.8%)	3 (17.7%)	5 (29.4%)	0
**13. Graphic designer**	4 (23.5%)	2 (11.8%)	1 (5.9%)	**9 (52.9%)**	2(11.8%)	0
**14. HR managers**	**9 (52.9%)**	5 (29.4%)	4 (23.5%)	1 (5.9%)	4 (23.5%)	1
**15. Inspectorate of Social Affairs and Employment**	2 (11.8%)	**7 (41.2%)**	4 (23.5%)	3 (17.7%)	5 (29.4%)	1
**16. Labour lawyer**	2 (11.8%)	**7 (41.2%)**	1 (5.9%)	3 (17.7%)	5 (29.4%)	1
**17. Lifestyle coach**	4 (23.5%)	2(11.8%)	3 (17.7%)	5 (29.4%)	4 (23.5%)	0

**18. Maintenance specialist (a specialist in maintaining the use of an eHealth technology)**	2 (11.8%)	5 (29.4%)	4 (23.5%)	3 (17.7%)	4 (23.5%)	0
**19. Marketing department**	2 (11.8%)	3 (17.7%)	1 (5.9%)	7 (41.2%)	4 (23.5%)	0
**20. Participation council within organizations**	**8 (47.1%)**	**7 (41.2%)**	2 (11.8%)	1 (5.9%)	2 (11.8%)	2
**21. Research team**	4 (23.5%)	**6 (35.3%)**	**7 (41.2%)**	1 (5.9%)	4 (23.5%)	2
**22. Organisations in eHealth design**	5 (29.4%)	3 (17.7%)	2 (11.8%)	4 (23.5%)	4 (23.5%)	0
**23. Programmer/software developer**	3 (17.7%)	4 (23.5%)	4 (23.5%)	**6 (35.3%)**	2 (11.8%)	0
**24. Product owner**	**8 (47.1%)**	3 (17.7%)	3 (17.7%)	3 (17.7%)	4 (23.5%)	1
**25. Self-tracking device develope**	5 (29.4%)	3 (17.7%)	3 (17.7%)	4 (23.5%)	5 (29.4%)	0
**26. Usability specialist**	2 (11.8%)	2 (11.8%)	2 (11.8%)	**6 (35.3%)**	**6 (35.3%)**	**0**
**27. Employers**	**10 (58.8%)**	**6 (35.3%)**	5 (29.4%)	0	3 (17.7%)	2
**28. Employees**	3 (17.7%)	**7 (41.2%)**	**8 (47.1%)**	3 (17.7%)	2 (11.8%)	2
**29. Health insurer**	5 (29.4%)	**6 (35.3%)**	3 (17.7%)	3 (17.7%)	2 (11.8%)	1
